# High-Risk Human Papillomavirus Targets Crossroads in Immune Signaling

**DOI:** 10.3390/v7052485

**Published:** 2015-05-21

**Authors:** Bart Tummers, Sjoerd H. Van Der Burg

**Affiliations:** Department of Clinical Oncology, Leiden University Medical Center, Albinusdreef 2, 2333 ZA Leiden, The Netherlands; E-Mail: b.tummers@lumc.nl

**Keywords:** high-risk human papillomavirus, keratinocyte, immune evasion

## Abstract

Persistent infections with a high-risk type human papillomavirus (hrHPV) can progress to cancer. High-risk HPVs infect keratinocytes (KCs) and successfully suppress host immunity for up to two years despite the fact that KCs are well equipped to detect and initiate immune responses to invading pathogens. Viral persistence is achieved by active interference with KCs innate and adaptive immune mechanisms. To this end hrHPV utilizes proteins encoded by its viral genome, as well as exploits cellular proteins to interfere with signaling of innate and adaptive immune pathways. This results in impairment of interferon and pro-inflammatory cytokine production and subsequent immune cell attraction, as well as resistance to incoming signals from the immune system. Furthermore, hrHPV avoids the killing of infected cells by interfering with antigen presentation to antigen-specific cytotoxic T lymphocytes. Thus, hrHPV has evolved multiple mechanisms to avoid detection and clearance by both the innate and adaptive immune system, the molecular mechanisms of which will be dealt with in detail in this review.

## 1. Introduction

Human papillomaviruses (HPVs) are small, non-enveloped icosahedral viruses belonging to the *Papillomaviridae* family. HPV is widespread within all human populations and transmitted via the skin, including the genitalia. With a double-stranded episomal DNA genome of only 7–8 kb, containing six non-structural early genes (E6, E7, E1, E2, E4 and E5), and two late genes (L2 and L1) that encode the capsid proteins [1], HPVs induce diseases ranging from warts to cancers [2]. Over 150 HPV types have currently been identified. They are divided into genera α, β, γ, μ and ν, based on the nucleotide sequence of the L1 gene [3]. HPV types of the α genus (~40) infect cutaneous and mucosal epithelia. Based on their oncogenic potential, mucosal HPVs are classified as low-risk, associated with benign warts or epithelial lesions, or high-risk, that can cause oropharyngeal and anogenital malignancies, including cancers of the cervix, vulva, vagina, penis and anus. HPV types of the other genera infect cutaneous epithelium and are associated with cutaneous papillomas and warts. βHPV types can cause non-melanoma skin cancer in immunocompromised individuals [4]. Most HPV infections resolve spontaneously within one (70%) to two (90%) years [5], and in only <1% of cases malignancies develop. Still, HPV causes ~528,000 new cancer cases and ~266,000 deaths each year. High-risk HPV (hrHPV) types are responsible for ~5% of all human cancers and are detected in 99.7% of cervical cancer cases, the fourth most common cancer in women, accounting for 7.5% of all cancer-associated deaths in women worldwide per year [6,7]. HPV types 16, 18, 31, 33, 35, 39, 45, 51, 52, 56, 59, 69, 73 and 82 have been detected in cervical carcinomas, but HPV16 is the most prevalent hrHPV type in cervical cancer and dominant in all other HPV-induced cancers [8,9].

HPVs exclusively infect keratinocytes (KCs) of the basal layer of the epidermis and mucosal epithelia, which they reach via micro-wounds and abrasions. Binding of the L1 protein of HPV to heparan sulfate proteoglycans at the surface of KCs induces endocytosis of the virion. Subsequently the capsid disassembles following acidification of the endosome and then the viral episome, still associated with L2, travels via the Golgi apparatus and Endoplasmic Reticulum to the nucleus [10] where low levels of viral early proteins are produced that reside mainly in the nucleus [11]. E1 and E2 initiate episome replication and, together with the host DNA replication machinery, maintain a low episome copy-number of 50–100 per cell [12]. Furthermore, E6 and E7 are produced to prevent cell growth arrest and apoptosis and delay differentiation, by inactivating p53 and the retinoblastoma protein (pRB). This induces a proliferative, non-differentiating state of the infected KC, resulting in lateral cell division. As the infected KC differentiates and migrates through the suprabasal layers of the epithelium, the expression of all viral genes is induced to enhance viral episome replication, which reaches high copy-numbers of hundreds to thousands per cell. In the higher layers of the epithelium the production of the late proteins L1 and L2, together forming the viral capsid, is induced and virion assembly takes place. With the rupture and shedding of the matured KC the viral particles are released [13]. Sometimes, for yet unknown reasons, hrHPV genomes can spontaneously integrate into the host genome, leading to release of the tight regulation of E6 and E7 expression. The newly transformed cells stably express E6, which binds to p53 and recruits the E3 ligase E6AP to target p53 for proteasomal degradation, as well as E7, which recruits the E3 ligase cullin 2 to target pRb for proteasomal degradation. The loss of these tumor suppressors results in uncontrollable cell growth, host genome mutations and inhibition of apoptosis, ultimately leading to cancer formation [1,13,14]. 

High-risk HPV infections can persist despite viral activity in keratinocytes. This indicates that HPV has developed mechanisms to effectively evade or suppress the host’s innate and/or adaptive immune response. Indeed, several studies on the spontaneous immune response to HPV have shown that HPV-specific cellular immunity develops quite late during persistent HPV infections and often are of dubious quality in people with progressive infections [15]. 

Viral persistence may be linked to the life cycle of HPV since HPV does not cause viremia, cell death, or cell lysis, and the life-cycle takes place within the boundary of the *lamina* *basalis*, away from dermal immune cells. Thus, spontaneous contact between the immune system and the virus are minimal and inflammatory responses are not readily elicited. Langerhans cells residing within the epidermis can sense viral presence, but HPV counteracts their recruitment by interfering with the production of immune attractants. In addition, after the infection is established in basal keratinocytes, major viral gene expression is differentiation dependent and as such viral peptide presentation to immune cells is limited. Besides these passive mechanisms to evade the immune system, hrHPV also actively interferes with innate and adaptive immune mechanisms. These are discussed below. 

## 2. Viral Recognition by Keratinocytes

Keratinocytes are well equipped to sense pathogens. Basal KCs express pattern-recognition receptors (PRRs), such as Toll-like receptors (TLRs), NOD-like receptors (NLRs), and RNA helicases, to recognize pathogen-associated molecular patterns (PAMPs) on viruses and microbes. PRR ligation leads to activation of inflammatory and proliferative signaling cascades and subsequent production of pro-inflammatory cytokines that can induce innate and adaptive immune responses. *In vitro* studies showed that KCs express TLR 1, 2, 3, 5 and 6 on the cell-surface and the nucleic acid-sensing TLR3 in endosomes. TLR7 and TLR8 are not expressed, but TLR7 expression can be induced upon TLR3 ligation [16]. The expression of TLR4 and TLR9 in basal KCs is still under debate, but TLR9 expression can be induced after terminal differentiation [17]. Cytosolically, KCs express the RNA helicases retinoic acid-inducible gene I (RIG-I; DDX58) and melanoma differentiation-associated protein 5 (MDA5; IFIH1) [18], and the dsDNA sensors gamma-interferon-inducible 16 (IFI16) and absent in melanoma 2 (AIM2) [19]. Expression of TLR3, 7, 9, PKR, RIG-I and MDA5 was confirmed *in situ* [18,20]. 

Although the vesicle-mediated entry mechanism used by HPV may hide the virus from recognition by cytoplasmic DNA sensors, KCs can produce type I interferon (IFN) and pro-inflammatory cytokines upon viral entry and, therefore, do recognize HPV [17]. Indeed, the episome contains CpG motifs that can be recognized by TLR9 [21] and the viral capsid itself is a potential PAMP. Whether HPV interferes with the expression of TLRs, RIG-I or MDA5 in HPV episome-containing KCs is still under debate [17,18,22]. While *TLR9* expression and function was shown to be abolished in KCs that overexpressed HPV16 E6 and E7 [21], by an E7-induced recruitment of a NFκB1, ERα and HDAC1 inhibitory complex to the TLR9 promotor [23], others concluded that E6 nor E7 influenced TLR9 expression or function [24]. The DNA sensor AIM2 is strongly expressed in HPV16-infected skin lesions, whereas IFI16 expression is not elevated [19]. Hence, it is not yet clear if HPV affects the expression of virus sensory molecules on KCs. 

## 3. HPV Influences Innate Immune Signaling

Keratinocytes produce type I IFNs and pro-inflammatory cytokines upon PRR ligation through signaling via interferon regulatory factor (IRF) and nuclear factor of kappa-light-chain-enhancer of activated B cells (NFκB) activating pathways. Type I IFNs (mainly IFNα (13 subtypes) and IFNβ, but also IFNε, IFNτ, IFNκ, IFNω, IFNδ and IFNζ) stimulate cells to express genes inducing an anti-viral state. They can also stimulate dendritic cells and as such act as a bridge between innate and adaptive immunity [25,26,27]. Pro-inflammatory cytokines are chemoattractants for immune cells and regulate cell migration, activation, polarization and proliferation. Several genome-wide transcription studies reported that hrHPV types 16, 18 and 31 influence—mainly reduce—basal, TLR3-induced cytokine expression, and type I IFN-induced interferon-stimulated gene (ISG) expression [18,28,29,30], indicating that hrHPV affects PRR- and type I IFN-induced signaling pathways. 

### 3.1. The Effect of HPV (Proteins) on the IRF Signaling Pathway

**Figure 1:**
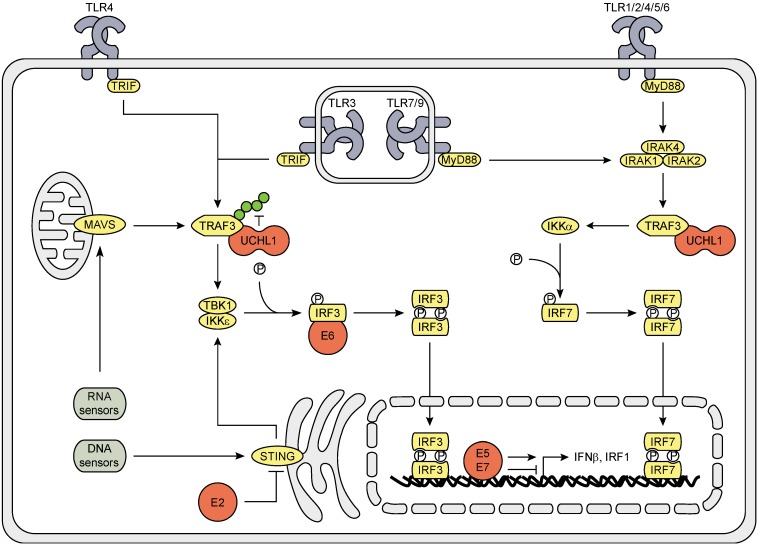
Schematic representation of the effects of hrHPV on IRF signaling. All TLRs, except TLR3, activate IRF7 via signaling through MyD88, the IRAK complex, TRAF3 and IKKα. TLR3 and 4 signal via TRIF, cytosolic RNA sensors through MAVS and cytosolic DNA sensors via STING activate IRF3 through TRAF3, TBK1 and IKKε. Activated IRFs dimerize, translocate to the nucleus and initiate gene transcription. HPV utilizes its own encoded E proteins (red) as well as exploits the cellular protein UCHL1 (red) to interfere with these signaling pathways. Green circles on TRAF3 indicate K63-linked poly-ubiquitin chains.

All TLRs, except TLR3, convey their signals via the adapter molecule MyD88. This induces the IRAK complex (consisting of IRAK1, 2 and 4) to recruit TRAF3, which stimulates IKKα to phosphorylate IRF7. TLR3 and 4 signal via TRIF, cytosolic RNA sensors via MAVS, and cytosolic DNA sensors signal via the adaptor molecule STING to activate TRAF3, which then induces the TBK1-IKKε complex to phosphorylate IRF3. Phosphorylated IRF3 and IRF7 homo-dimerize and translocate to the nucleus where production of type I IFNs is initiated. Furthermore, PRR ligation can result in IRF1 activation (Figure 1).

HrHPV influences type I IFN production by interfering at several points in the signaling cascade. HrHPV E2 proteins reduce the expression of *STING* and *IFNκ* [31], the latter of which its expression is also reduced by E6 [22,32]. HPV16, but not HPV18, E6 protein binds to IRF3 and, thereby, may prevent its transcriptional activity [33]. E7 blocks *IFNβ* transcription by binding to IRF1 and recruiting histone deacetylases (HDACs) to the *IFNβ* promotor site [34,35]. In contrast, E5 enhances *IFNβ* and *IRF1* expression [36]. HrHPV also exploits cellular proteins to interfere with IRF signaling; it upregulates the endogenous deubiquitinase ubiquitin carboxy-terminal hydrolase L1 (UCHL1) to interact with and deubiquitinate K63-linked poly-ubiquitin chains from TRAF3, resulting in reduced TBK1—TRAF3 interaction, IRF3 phosphorylation and *IFNβ* expression [17]. 

### 3.2. The Effects of HPV (Proteins) on IFNAR Signaling

**Figure 2:**
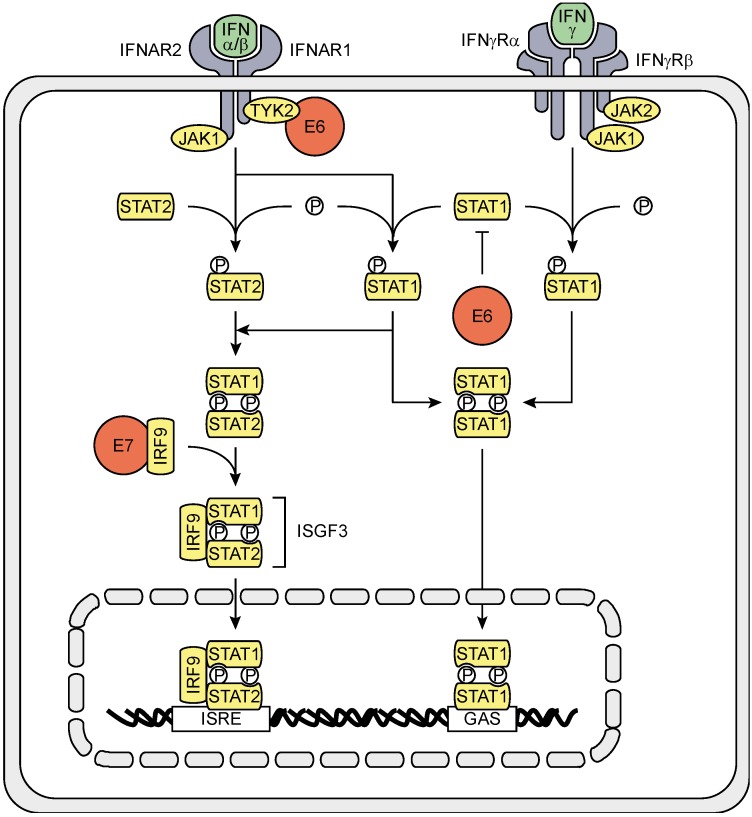
Schematic representation of the effects of hrHPV on IFNAR and IFNγR signaling. Type I IFN binding to the IFNAR leads to signaling via JAK1 and TYK2 to activate STAT1 and STAT2. STAT1 and STAT2 heterodimerize and recruit IRF9, forming the ISGF3 complex, which translocates to the nucleus, binds to ISREs and initiates ISG transcription. Activated STAT1 can also homodimerize, translocate to the nucleus, bind to GAS and initiate ISG transcription. Type II IFN binding to the IFNγR results in the activation of JAK1 and JAK2 and recruitment and phosphorylation of STAT1, which homo-dimerizes, translocates to the nucleus, binds to GAS on the DNA and initiates ISG transcription. HPV proteins (red) interfere with both IFNAR and IFNγR signaling by decreasing STAT1 levels, and hampering TYK2 and IRF9.

The PRR-induced type I IFNs IFNα and IFNβ are secreted and can induce IFN-stimulated gene (ISG) expression in the infected cell itself but also in their uninfected neighbors. IFNα and IFNβ bind to the heterodimeric transmembrane IFNα/β receptor (IFNAR), composed of the IFNAR1 and IFNAR2 subunits. The IFNAR activates the receptor-associated protein tyrosine kinases Janus kinase 1 (JAK1) and tyrosine kinase 2 (TYK2), which recruit and phosphorylate STAT1 and STAT2, causing them to hetero-dimerize, bind IRF9, thereby forming the IFN-stimulated gene factor 3 (ISGF3) complex, and translocate to the nucleus. ISGF3 binds to IFN-stimulated response elements (ISREs) on the DNA and activates ISG transcription. IFNAR ligation can also lead to STAT1 homo-dimerization. STAT1 homo-dimers translocate to the nucleus and bind to γ-activated sequences (GAS) on the DNA, thereby activating ISG transcription more associated with IFNγ signaling (Figure 2) [26,37]. 

HrHPV also interferes with IFNAR signaling. HPV18 E6 can bind to TYK2 in order to hamper phosphorylation of STAT1 and STAT2 [38]. E6, and to a lesser extend E7, of the hrHPV types 16 and 31 were shown to impair STAT1 transcription and translation, and binding of STAT1 to the ISRE [28,30,39]. However, although hrHPV represses STAT1 protein levels, the IFNβ-induced STAT1 signal cascade is not affected by hrHPV, as phosphorylation of STAT1 still occurs [39]. Expression of STAT2 and IRF9 are not affected, but E7 can interact with cytosolic IRF9, preventing IRF9 to translocate to the nucleus with as a consequence impairment of ISGF3 complex formation [40,41].

### 3.3. The Effect of HPV (Proteins) on the NFκB Signaling Pathway

**Figure 3:**
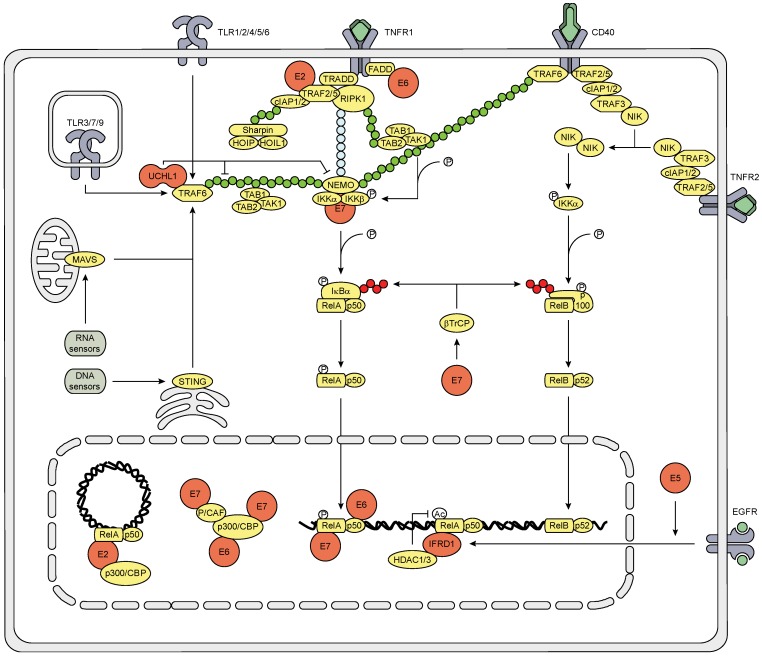
Schematic representation of the effects of hrHPV on NFκB signaling. The canonical NFκB1 pathway is activated by PRRs and CD40 through TRAF6 and TNFR1 through RIP1. Poly-ubiquitination of TRAF6 and RIP1 recruits the TAB1-TAB2-TAK1 and IKK complexes resulting in the phosphorylation of IKKβ by TAK1. IKKβ phosphorylates IκBα, which is then ubiquitinated by SCF-βTrCP and subsequently degraded, and thereby releases the NFκB1 complex to translocate to the nucleus. CD40 and TNFR2 initiate non-canonical NFκB2 signaling by recruitment of TRAF2/5, cIAP1/2 and TRAF3 to the respective receptor, leading to TRAF3 degradation. This causes NIK to accumulate and activate IKKα to phosphorylate p100. This induces SCF-βTrCP to ubiquitinate p100, leading to the proteosomal processing of p100 into p52, and the subsequent nuclear translocation of NFκB2. In the nucleus NFκB binds to the DNA and is aided by coactivators to initiate gene transcription. HPV utilizes its own encoded E proteins (red) as well as exploits the cellular proteins (red) UCHL1 and IFRD1 to interfere with NFκB1 signaling at multiple positions in the pathway. Green circles indicate K63-linked poly-ubiquitin chains, red circles indicate K48-linked poly-ubiquitin chains, and blue circles indicate linear poly-ubiquitin chains.

PRRs also induce cytokine production through signaling via TRIF, MAVS, STING and the IRAK complex, which leads to the K63-linked poly-ubiquitination of TRAF6. The TAB1-TAB2-TAK1 complex and the IKK complex (consisting of NEMO, IKKα and IKKβ) bind to the poly-ubiquitin chain on TRAF6, resulting in the phosphorylation of IKKβ by TAK1. Activated IKKβ then phosphorylates IκBα, leading to the SCF-βTrCP-mediated K48-linked poly-ubiquitination of IκBα and its subsequent degradation. This releases the NFκB1 complex (consisting of RelA and p50) and allows it to translocate to the nucleus where it is further modified to induce DNA binding and transcriptional activation (Figure 3) [27,42].

By the use of several different model systems hrHPV or its individual proteins have been shown to affect the PRR-induced signaling cascade that leads to NFκB nuclear translocation and to impair the function of NFκB within the nucleus. HrHPV upregulates the NFκB family members RelA, c-Rel, and the precursor proteins p105 and p100, which are processed into p50 and p52, respectively, and sequesters these proteins in the cytoplasm [30,43,44,45,46]. The last NFκB family member, RelB, is not reported to be regulated by HPV. HrHPV exploits the endogenous protein UCHL1 to bind TRAF6 and influence the Ub status of TRAF6 and NEMO, resulting in NEMO degradation [17]. Furthermore, UCHL1 can prevent IκBα ubiquitination [47]. 

Within the nucleus, E6 reduces NFκB RelA-dependent transcriptional activity [48], by binding to the C/H1, C/H3 and C terminal domains of CBP/p300 [49,50], thereby competing with RelA and SRC1, which bind the C/H1 and C terminal domain of CBP/p300, respectively [51]. P/CAF can still bind to the C/H3 domain of CBP/p300 in presence of E6, but P/CAF cannot acetylate NFκB since E7 binds to, and thereby blocks, the HAT domain of P/CAF [51]. E7 blocks NFκB DNA binding activity [35] and competes with E2 for binding the C/H1 domain of p300/CBP, thereby hampering E2 transactivation [52]. In contrast, E2 binds to p300/CBP [53,54] and increases NFκB signaling by enhancing RelA expression and transcriptional activation upon TNFα treatment [45]. 

HrHPV upregulates EGFR gene and surface expression via the E5, E6 and E7 proteins [55,56], and enhances EGFR signaling via E5 and E6 [57,58,59]. EGFR activation on epithelial cells has been shown to result in a decreased production of pro-inflammatory cytokines [60,61,62]. HrHPV-induced EGFR signaling, via mTOR, RAF and/or MEK1, increases the expression of the cellular protein interferon-related developmental regulator 1 (IFRD1) which mediates RelA K310 deacetylation by HDAC1/3 and, thereby, attenuates the transcriptional activity of NFκB1 [56]. 

### 3.4. The Effect on the Inflammasome Pathway

It is not clear whether the inflammasome pathway is important in the protection against HPV. However, recently it was reported that the production of IL1β, a cytokine that is secreted upon cleavage of pro-IL1β by inflammasome-activated caspase1, is impaired. HPV E6 binds to E6-AP and p53 and this complex induces the inflammasome-independent proteasome-mediated degradation of pro-IL1β and, as such, hampers IL1β formation [63], indicating that hrHPV may suppress immunity by interference with post-translational processes. 

Altogether it is clear that hrHPV invested heavily in preventing infected cells to adapt an anti-viral state as well as to suppress the production of cytokines that can induce the attraction of adaptive immune cells which may control HPV infection.

## 4. The Action of KCs to Secondary Immune Signals is Suppressed by HPV

Cells of the adaptive immune system, in particular T cells, are activated by APCs in the lymph nodes and migrate to infected sites. They produce cytokines and express ligands that can activate signaling cascades in the KC involved in survival and pro-inflammatory cytokine production, leading to killing of KCs and in parallel the reinforcement of adaptive immunity. Despite the infiltration of adaptive immune effector cells the persistence of hrHPV-infected sites suggests that hrHPV has evolved mechanisms to resist this attack. CD4^+^ T helper 1 (Th1) cells are especially important in controlling hrHPV infections. However, even vaccines that boost viral Th1 immunity during chronic infection are only partially successful [64]. Th1 cells produce IFNγ and TNFα, and express CD40L, which induce cytokine production and proliferative changes in KCs. 

TNFα is the ligand for both the TNFα receptor 1 (TNFR1) and TNFR2. TNFR1 activates canonical NFκB1 by recruiting and activating TRADD, leading to the formation of a complex consisting of RIP1, TRAF2 or 5, and cIAP1 or 2. cIAP1/2 is ubiquitinated with a K63-linked poly-ubiquitin chain to which the LUBAC complex (consisting of Sharpin, HOIP and HOIL1) binds. RIP1 is ubiquitinated with both K63-linked and linear poly-ubiquitin chains. The TAB1-TAB2-TAK1 complex binds to the K63-linked poly-ubiquitin chain and phosphorylates the IKK complex that binds to the linear poly-ubiquitin chain of RIP1, leading to NFκB1 release through IKKβ-induced SCF-βTrCP-mediated degradation of IκBα. TNFR2 activates the non-canonical NFκB2 pathway by recruiting TRAF2/5, cIAP1/2 and TRAF3, resulting in TRAF3 degradation. This abrogates TRAF3-induced NIK degradation, causing NIK to accumulate and activate IKKα. IKKα phosphorylates the p100 NFκB precursor protein of the NFκB2 complex, which further consists of RelB. This induces SCF-βTrCP to ubiquitinate p100 with a K48-linked poly-ubiquitin chain, leading to the proteosomal processing of p100 into p52, and the subsequent nuclear translocation of the p52-RelB dimer (Figure 3). 

HPV interferes with these cascades in a similar way as it attenuates PRR-induced NFκB signaling by using its own E proteins and endogenous proteins. Additionally, E6 binds to the C terminus of TNFR1 [65], and the N terminus of the death effector domains (DEDs) of FADD, which accelerates the degradation of FADD [66], thereby hampering the induction of apoptosis. E6 does not bind to the TRADD adaptor molecule [66]. Furthermore, E7 binds to the IKK complex and attenuates TNFα-induced kinase activity of IKKα and IKKβ, which hampers IκBα phosphorylation and degradation, and subsequent NFκB nuclear translocation [48]. In contrast to E6, E7, UCHL1 and IFRD1, E2 stimulates TNFα-induced, but not IL1-induced, NFκB signaling [45,67], by directly interacting with TRAF5 and TRAF6, but not TRAF2, thereby stimulating K63-linked ubiquitination of TRAF5 [67]. 

IFNγ and TNFα are known to synergistically affect gene expression, and also in KCs pro-inflammatory cytokine expression is synergistically higher than expression induced by IFNγ or TNFα alone. Still, hrHPV attenuates IFNγ and TNFα-induced pro-inflammatory cytokine expression and the attraction of PBMCs to KCs that have been stimulated with the combination of IFNγ and TNFα [56]. Ligation of the IFNγR with type II IFN results in the activation of JAK1 and JAK2 and recruitment and phosphorylation of STAT1, which homo-dimerizes, translocates to the nucleus, binds to GAS on the DNA and initiates ISG transcription (Figure 2). The effects of HrHPV on the IFNγ-signaling pathway might be explained by the repressed STAT1 expression and protein levels in HPV infected cells, albeit that STAT1 phosphorylation still is intact [39,68]. However, exposure of hrHPV-infected KCs to IFNγ fails to induce cellular programs associated with a block of proliferation [68]. Furthermore, IFNγ and TNFα stimulation induces processing of the non-canonical NFκB precursor p100 into p52 in hrHPV-infected cells but not uninfected KCs (Tummers, Unpublished data), indicating that hrHPV skews the response of KCs upon stimulation with TNFα and IFNγ towards the non-canonical NFκB pathway. Potentially, this is caused by E7 as this oncoprotein was shown to increase SCF-βTrCP protein levels [69] and in this way might accelerate IκBα degradation and p100 processing [70]. Although unexplored at this point, it is highly likely that this forms another pathway allowing hrHPV-infected cells to resist control of infection by the immune system. Last but not least, epithelial cells express CD40 on their cell surface [71]. Ligation of CD40 induces both canonical and non-canonical NFκB activation, similar to TNFR1 and 2, respectively. Activation of this pathway in epithelial cells results in a very coordinated response by KCs, dominated by the expression of genes involved in leukocyte migration, cell-to-cell signaling and interaction, as well as cell death and survival. The presence of HPV does not affect the gene expression profile of CD40 stimulated KCs, but it does attenuate the extent of the response and reduces the attraction of PBMCs [72]. Based on our previous studies it is likely that the CD40—NFκB1 axes of CD40 signaling is affected via the interaction of UCHL1 and TRAF6, the effects of E7 on the IKK complex, and that of IFRD1 on NFκB1 transcriptional activation. Speculatively, at the non-canonical side signaling could be hampered by abrogation of UCHL1-mediated TRAF2 and/or 5- or E7-mediated IKKα functioning. However, UCHL1-mediated TRAF3 hampering could also lead to constitutive NIK accumulation and subsequent pathway activation. It remains to be determined if hrHPV prefers to skew KCs towards non-canonical NFκB activation after CD40 ligation. 

In conclusion, hrHPV does not only try to prevent the attraction of immune cells via the impairment of cytokine secretion but it also hampers the regulation of intracellular growth and apoptosis programs of infected cells that normally are activated as a response to effector molecules of the adaptive immune system.

## 5. HrHPV Influences MHC Surface Expression and Peptide Presentation

**Figure 4:**
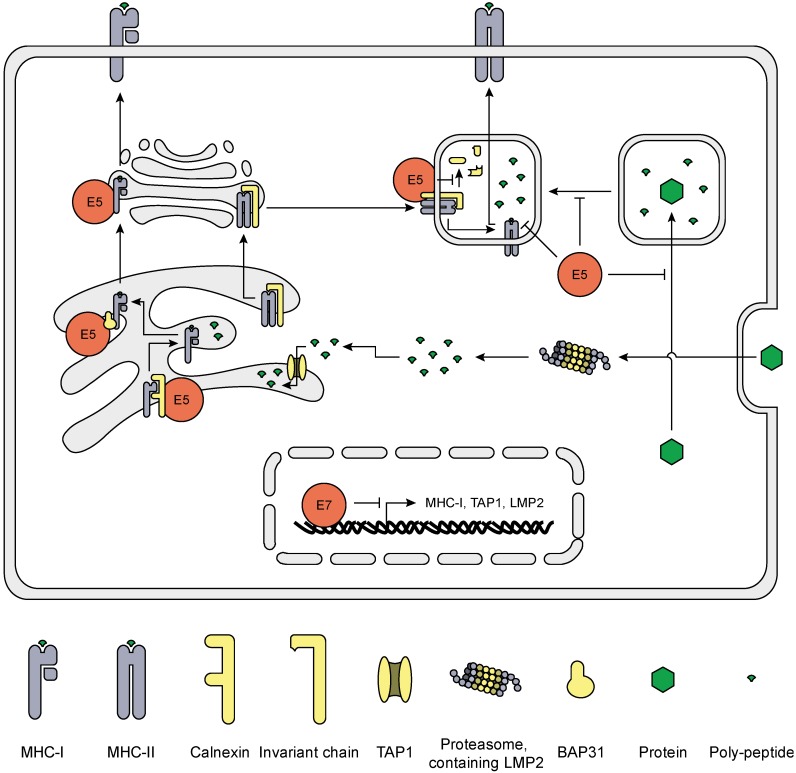
Schematic representation of the effects of hrHPV on antigen presentation. The proteasome processes proteins into peptides, which are transported into the ER via TAP1. Aided by several chaperone proteins, MHC-I is folded and loaded with peptide after which it exits the ER to travel via the Golgi apparatus to the plasma membrane were the peptides are presented to T cells. HPV proteins (red) attenuate gene expression of critical components of this pathway, as well as actively retains MHC-I in the ER and Golgi apparatus. MHC-II forms in the ER and complexes with the invariant chain. The complex travels via the ER and Golgi apparatus to lysosomes where the invariant chain is degraded and MHC-II is loaded with processed peptides from endocytosed proteins. Loaded MHC-II then travels to the plasma membrane to present the peptides. Upon IFNγ stimulation, HPV E5 (red) blocks invariant chain degradation and peptide loading, as well as inhibits endosome acidification and maturation.

The attack of virus-infected cells by T cells is a highly effective and specific mechanism to prevent the production and spread of virus particles. T cells recognize cells when viral protein-derived peptides are presented in the context of MHC molecules. Literature shows that primary KCs constitute excellent targets for antigen-specific cytotoxic T lymphocytes (CTLs) if their cognate peptide is presented on the KCs cell surface [73]. The overexpression of E5 [74] or E7 [75], however, makes cells more resistant to CTL-mediated lysis. E5 and E7 both reduce MHC-I surface expression, but act on different levels (Figure 4). E7 reduces MHC-I gene expression by physically associating with a putative RXRbeta binding motif (GGTCA) of the proximal promoter of MHC-I genes and recruiting HDAC1, 2 and 8 to this promoter site, leading to repressed chromatin activation. Indeed, E7 knock-down in Caski cells released HDAC1 and 2 from the MHC class-I promoter, and increased histone acetylation and MHC-I expression [75,76,77,78,79]. Furthermore, E7 represses the LMP2 and TAP1 promotors [76,77], two important proteins involved in peptide production and transportation, respectively. E7 also reduces IRF1 expression by suppression of IFNγ-induced STAT1-Tyr701 phosphorylation, repressing IFNγ-mediated upregulation of MHC-I expression via the JAK1/JAK2/STAT1/IRF-1 signal transduction pathway [80,81]. E5 does not influence MHC-I synthesis, but reduces MHC-I surface expression [80] by retaining MHC-I in the Golgi complex via interaction of di-leucine motifs (LL1 and LL3) localized in the N-terminal helical transmembrane (TM1) region of the protein [82]. This E5—MHC-I interaction is not haplotype specific, suggesting that E5 can hamper all MHC-I-dependent antigen presentation [83]. Moreover, binding of the TM1 domain of E5 to the ER chaperone Calnexin retains MHC-I in the ER [84], and down-regulates surface expression of CD1d, a sentinel protein in bridging innate and adaptive immunity [85]. Furthermore, via its C-terminus E5 can bind the B-cell-associated protein 31 (Bap31) [86], a protein involved in the exit of peptide-loaded MHC-I from the ER [87]. Interestingly, E5 selectively downregulates the surface expression of HLA-A and -B, but not that of HLA-C and HLA-E [80]. Under normal conditions expression of HLA class II is not affected but upon IFNγ stimulation E5 does abrogate MHC-II surface expression and blocks peptide-loading of MHC-II and invariant chain degradation [88], by inhibiting endosome acidification [89] or perturbing trafficking from early to late endocytic structures [90]. 

Thus, as a last resort, to evade host immunity HPV perturbates the expression of HLA class I and II molecules making the infected cells less visible to the adaptive immune system, slowing down the resolution of infected lesions.

## 6. Final Comments

Keratinocytes are well equipped to recognize and react to invading pathogens, and hrHPV is no exception to this. However, hrHPV initiates several immune evasion mechanisms soon after infecting the KC. The virus interferes with the innate immune response by affecting several signaling pathways that otherwise would prompt anti-viral mechanisms in the host cell. Furthermore, hrHPV interferes with the production of cytokines that are involved in the attraction of immune cells to the infected epithelium. In addition, the virus hides itself from the immune system by suppressing the antigen presentation machinery normally allowing infected cells to be recognized by adaptive immune cells and, if this is not successful, hrHPV still employs means to hamper the response of KC’s to signals from the effector molecules used by adaptive immune cells to exert their antiviral function. 

The IFN pathway seems to be centrally attacked through downregulation of STAT1 levels which is observed in hrHPV episome-bearing KCs when compared to uninfected KCs. Downregulation of STAT1 results in attenuated ISG expression, albeit that signaling downstream of the IFNAR and IFNγR still functions [39,68]. Thus, the attenuated type I IFN-induced ISG expression in HPV+ KCs must be due to the basal lowered STAT1 levels. In contrast, in experiments where E6 is overexpressed, E6 was shown to bind TYK2 and to interfere with STAT1 and STAT2 phosphorylation. In addition, overexpressed E7 binds and sequesters IRF9 in the cytosol, so that the ISGF3 complex cannot form in the nucleus. Blocking the type I IFN response is also beneficial for the virus as it allows viral replication. Long-term high-dose IFNβ treatment of HPV-episome bearing KCs results in growth arrest and apoptosis [91,92], thus preventing viral replication. Treatment of KCs with IFN upregulates *IFIT1* (*ISG56*), which can block E1-mediated episome replication by directly interacting with E1, inhibiting E1 DNA helicase activity and causing E1 to translocate from the nucleus to the cytosol [93]. By interfering with IFN signaling through downregulation of STAT1 HPV is able to maintain and amplify its episomes [39]. Interestingly, in virally infected cells p53 was shown to boost type 1 IFN production and signaling resulting in enhanced apoptosis of the infected cells with as consequence limited spread of the infection [94,95]. HPV interferes with the function of p53 and as such with the ability of KCs to boost their antiviral activity.

The canonical NFκB pathway is attacked by hrHPV at multiple positions in the signaling cascade downstream of immune receptors. This indicates that suppression of the NFκB pathway forms a very important target for the virus and implies that this pathway normally would allow the host to resist viral infection. There are several proteins involved in this process. Interestingly, E2 may promote canonical NFκB signaling. It may form an E2-NFκB-p300/CBP transcriptional repressor complex on the LCR of the episome and as such regulates episome transcription which is required for the virus to sustain a low profile. However, as luciferase assays show that the E2 protein renders NFκB more active, the virus thus may prompt E2-mediated NFκB-induced pro-inflammatory cytokine production and immune cell attraction, indicating that the virus needs additional mechanisms in order to regulate the episome while keeping pro-inflammatory cytokine expression in check during infection. Our knock-down experiments in HPV episome-bearing KCs revealed that hrHPV exploits the cellular proteins UCHL1 and IFRD1 to interfere with NFκB signaling. UCHL1 acts on TRAF proteins in the cytosol upstream of NFκB signaling, whereas IFRD1 attenuates the transcriptional activity of NFκB. The combined expression of E2, UCHL1 and IFRD1 during an infection thus might allow hrHPV to regulate its episome while suppressing KCs pro-inflammatory cytokine production (Figure 3). Furthermore, hrHPV seems to skew the response of KCs to IFNγ and TNFα towards the non-canonical NFκB pathway. How and why the virus does this is currently unknown, but it may be that hrHPV utilizes the non-canonical NFκB route to resist the anti-proliferative effects of IFNγ and TNFα [68]. 

In contrast to hrHPV-infected cells, higher intraepithelial neoplastic lesions and HPV-positive cancers often show overactive canonical NFκB gene expression [96]. Indeed, overexpression experiments showed that E6 and/or E7 can also have pro-NFκB signaling effects and can increase NFκB target gene expression [30]. Mechanistically, E6 targets the NFκB repressor NFX1-91 for degradation [97] and under hypoxic conditions hampers CYLD, a negative regulator of NFκB signaling [98]. E6 also upregulates gene expression of the NFκB signaling components p50, NIK and TRAIP [30]. E7 upregulates SCF-βTrCP protein levels [69], which might lead to accelerated IκBα degradation and p100 processing. The transformed cell may benefit from E6/E7-enhanced NFκB signaling by maintaining a proliferative, anti-apoptotic state, although also pro-inflammatory cytokine expression is increased. Notably, cell type and growth rate are important determinants whether HPV E6 or E6/E7 stimulate or inhibit NFκB activation [99]. 

Most HPV infections resolve spontaneously, although HPV invests heavily in suppressing host immunity. This indicates that external factors, such as genetic and environmental factors may contribute to the establishment of a persistent infection and progression to cancer. Genetic predisposition to cervical tumors was found [100] and several combinations of single nucleotide polymorphisms (SNPs) were associated with an increased risk to cancer. SNPs in genes of the antigen processing machinery, such as HLA-A, LMP7, TAP2 and ERAP1 [101], and in the FANCA and IRF3 genes [102] were linked to persistent HPV infection and formation of cancer. SNPs in the TLR and NFκB pathways were also studied [103]. Of the thirty-two candidate genes involved in these pathways, including TLR3, NFκB1, NFκB2, RelA, RelB, TRAF3 and TRAF6, only a SNP in the 5' UTR of the lymphotoxin alpha (LTA; TNF superfamily member 1) was significantly associated with increased risks of cervical and vulvar cancers [103]. Based on the interactions between the different proteins in the downstream signaling pathways and their outcomes with respect to activation, splicing, degradation and translocation it might well be that combinations of SNPs, of multiple genes associated with the IRF and NFκB pathways, rather than single SNPs, may confer protection or susceptibility towards persistence of HPV infection and the ultimate progression to cancer.

In conclusion, there is accumulating evidence that hrHPV targets multiple immune-associated pathways. Notably, since viral gene expression considerably differs between hrHPV-infected KCs and hrHPV-transformed cells, data obtained from viral protein overexpression experiments should be carefully interpreted with respect to what their effects are in infection or cancer. 
